# The (p)ppGpp Synthetase RelA Contributes to Virulence, Competition Capability and Antibiotic Resistance of Avian Pathogenic *Escherichia coli*

**DOI:** 10.3390/microorganisms14081803

**Published:** 2026-08-16

**Authors:** Jiangang Hu, Dossêh Jean Apôtre Afayibo, Chang Liu, Mengjie Guo, Beibei Zhang, Weiqi Guo, Xinyu Wang, Lei Deng, Yanqing Bao, Jingjing Qi, Mingxing Tian, Shaohui Wang

**Affiliations:** Shanghai Veterinary Research Institute, Chinese Academy of Agricultural Sciences, 20241 Shanghai, China; vethjg@163.com (J.H.); jeanafayibo@gmail.com (D.J.A.A.); lc2558678213@163.com (C.L.); 18835497365@163.com (M.G.); bzhang1997@sina.com (B.Z.); gwq981124@163.com (W.G.); wangxinyu1823@163.com (X.W.); ldeng@shvri.ac.cn (L.D.); ybao@shvri.ac.cn (Y.B.); qijingjing@shvri.ac.cn (J.Q.); tianmx530@126.com (M.T.)

**Keywords:** avian pathogenic *Escherichia coli*, *relA*, stringent response, interbacterial competition, virulence, membrane integrity

## Abstract

Avian pathogenic *Escherichia coli* (APEC) induces avian colibacillosis and brings huge economic losses to global poultry production. The small alarmone (p)ppGpp mediates the bacterial stringent response, a vital pathway modulating microbial stress adaptation and pathogenic capacity. The functions of the (p)ppGpp synthase gene *relA* in APEC pathogenesis remain poorly characterized. In this study, we constructed a *relA* deletion mutant (ΔrelA) and its complemented strain (CΔrelA). The phenotypic and pathogenic characteristics of these strains were investigated. The results showed that deletion of *relA* did not significantly affect bacterial growth or motility. However, the ΔrelA strain showed increased susceptibility to aminoglycoside antibiotics. Furthermore, the enhanced interbacterial competition of the mutant was associated with the upregulation of core genes in the type VI secretion system (T6SS). Importantly, *relA* was essential for APEC adhesion to and invasion of avian DF-1 cells, as well as for colonization and virulence in ducklings, where ΔrelA exhibited significantly attenuated infectivity and reduced bacterial loads in the liver and spleen. Furthermore, transcriptomic analysis revealed that RelA deletion downregulated genes involved in integral components of the membrane, and further assays confirmed compromised membrane integrity in the mutant strain. These findings suggest that RelA maintains membrane integrity, which underpins its contributions to antibiotic resistance and virulence. These findings indicate that *relA* plays a key role in APEC virulence, antibiotic resistance, and membrane homeostasis, and could provide a theoretical basis for targeting the stringent response as a potential strategy to control avian colibacillosis.

## 1. Introduction

Avian pathogenic *Escherichia coli* (APEC) is the primary causative pathogen of avian colibacillosis, a systemic infectious disease that has continuously caused massive economic losses to the global poultry industry [1]. APEC infection manifests as various clinical conditions, including respiratory tract lesions, septicemia, and fatal systemic disease, and causes reduced yield of poultry products, posing major risks to animal health and food safety [1]. The pathogenesis of APEC involves adhesion, invasion, immune evasion, and intracellular survival. These processes require the bacterium to adapt to the stressful conditions of the host environment [2].

Bacteria encounter diverse environmental stresses that must be efficiently sensed and countered to ensure survival [3]. The small molecule alarmone (p)ppGpp mediates bacterial adaptive responses by modifying the initiation properties of RNA polymerase and is synthesized in Escherichia coli by two functionally related enzymes, RelA and SpoT [4]. The stringent response (SR), a central stress response pathway, is governed by these two homologous enzymes [5]. During amino acid starvation, the lack of aminoacyl-tRNAs activates ribosome-bound RelA, which constitutes the main source of (p)ppGpp synthesis [6]. Beyond mediating nutritional stress responses, (p)ppGpp contributes to virulence, host infection survival, antibiotic resistance, and persister cell formation [7]. Early research has demonstrated that (p)ppGpp, the core effector molecule of the bacterial stringent response, modulates bacterial growth rate and survival under multiple stress conditions, including nutrient limitation, exposure to antimicrobial compounds, and osmotic stress [7,8].

RelA is a monofunctional long RelA-SpoT Homolog (RSH) enzyme that synthesizes (p)ppGpp in response to amino acid starvation. Upon detecting uncharged tRNA in stalled ribosomes, RelA undergoes a conformational change that activates its synthetase domain, catalyzing the transfer of pyrophosphate from ATP to GDP [9]. (p)ppGpp promotes bacterial adhesion by activating key colonization factors. In enterohemorrhagic *E. coli*, RelA-mediated (p)ppGpp accumulation triggers the locus of enterocyte effacement (LEE) pathogenicity island via transcriptional activators ler/pch, enhancing epithelial adherence while downregulating flagellar genes [10,11]. In uropathogenic *E. coli*, (p)ppGpp induces type 1 fimbriae expression by activating the recombinase FimB [12].

The stringent response facilitates bacterial invasion via (p)ppGpp. In *Salmonella* Typhimurium, (p)ppGpp-null mutants show reduced intestinal epithelial invasion and attenuation in mice due to decreased SPI1 activators HilA/InvF expression, impairing type III secretion system function [13]. In addition, the (p)ppGpp deficiency reduces systemic infection, flagella production, and survival in phagocytes [14]. Beyond its roles in previously discussed strains, (p)ppGpp is also required for the invasion and virulence of multiple other intracellular pathogens, including *Campylobacter jejuni*, *Streptococcus suis*, *Legionella pneumophila*, and *E. faecalis* [9].

In chronic infections, (p)ppGpp drives antibiotic tolerance and resistance. A clinical methicillin-resistant *S. aureus* isolate from a chronic infection had an F128Y mutation in the Rel hydrolase domain, causing (p)ppGpp overexpression and constitutive stringent response activation. This upregulated the agr locus and yielded resistance to rifampicin, ciprofloxacin, and linezolid, a last-resort antibiotic, via mutations in RpoB, ParC, and RlmN [15]. The strain exhibited a slow-growing small colony variant phenotype, demonstrating that (p)ppGpp dysregulation enables emergence of highly resistant variants during chronic infection.

In addition, the contribution of the (p)ppGpp synthase gene *relA* in APEC pathogenesis is not fully characterized. In this study, we constructed a *relA* deletion mutant and its complemented strain using the APEC strain CE08. Elucidating RelA function may reveal the stringent response as a novel target for controlling avian colibacillosis.

## 2. Materials and Methods

### 2.1. Bacterial Strains, Plasmids and Cultural Conditions

All bacterial strains and plasmids used in this study are listed in Table 1. The wild-type APEC strain CE08 was initially isolated from a clinical case of avian colibacillosis. All of the test strains were cultivated in Luria–Bertani (LB) medium at 37 °C. When necessary, antibiotics such as ampicillin (Amp, 100 μg/mL) or chloramphenicol (Cm, 35 μg/mL) were supplemented into the medium. Ampicillin (100 μg/mL) was used for selecting transformants carrying pKD46 or pUC19, and chloramphenicol (35 μg/mL) was used for selecting strains carrying pKD3, pCP20, or pSTV28-derived plasmids.

### 2.2. Construction of Mutant and Complementation Strains

The *relA* gene deletion mutant strain was constructed using the λ Red recombinase system in accordance with the procedure described in previous studies [16]. All primers used in this study are listed in Table 2. Briefly, a linear DNA fragment harboring a chloramphenicol resistance cassette was electroporated into CE08 carrying pKD46, enabling allelic replacement of *relA*. The antibiotic marker was subsequently eliminated using pCP20, and chloramphenicol-sensitive colonies were screened. The resultant mutant was verified by PCR and sequencing and designated ΔrelA. For complementation, the *relA* coding sequence was amplified and cloned into pSTV28-Pamp. The recombinant plasmid pSTV28-relA was then introduced into the ΔrelA mutant, yielding strain CΔrelA. To confirm that the complemented strain appropriately expressed the *relA* gene, transcriptional validation was performed by qRT-PCR and supported by transcriptomic analysis, as described in Section 2.9 and Section 2.10.

### 2.3. Growth Curve and Motility Assays

Growth curves and motility phenotypes of CE08, ΔrelA, and CΔrelA were assessed in LB medium as detailed previously [17]. Briefly, cultures were shaken at 200 rpm and 37 °C, and cell density was recorded spectrophotometrically at 600 nm every hour over a 12 h period. For swimming and swarming assays, bacteria were spotted onto LB plates supplemented with 0.3% and 0.5% agar, respectively; halo diameters were quantified following overnight incubation at 37 °C. These assays were performed three times.

### 2.4. Determination of Minimum Inhibitory Concentration (MIC)

The protocol for MIC determination was adapted from a previously reported method [18]. MIC detection was performed via the broth microdilution method, with minor adjustments following the standards specified in the CLSI guidelines (CLSI M07-A11, 2018). All test strains were grown to an OD_600nm_ of 1.0, and the bacterial suspension was subsequently adjusted to an OD_600nm_ of 0.5 using fresh MH medium. In a 96-well plate, antibiotics were subjected to two-fold serial dilutions in MH medium. Each well, except the negative control, received 100 µL of the respective antibiotic dilution. Subsequently, 5 µL of the adjusted bacterial inoculum was added to all wells. For the negative control, each well received 5 µL of bacterial inoculum mixed with 195 µL of antibiotic-free MH broth. Following incubation at 37 °C for 24 h, the MIC value was defined as the minimal concentration at which no visible turbidity was observed. Each determination was carried out with three independent biological replicates.

### 2.5. Bacterial Competition Assays In Vitro

Bacterial competition assays were conducted as previously described, with minor modifications [19]. Fresh cultures of the donor (APEC) and recipient strains (DH5α) were each normalized to an OD_600nm_ of 0.5, and then mixed at a ratio of 5:1. The mixed cell suspension was spotted onto low-salt LB agar plates overlaid with nitrocellulose membranes, and incubated at 30 °C for 6 h. After co-culture, the bacterial spots were harvested, serially diluted, and inoculated onto LB agar plates supplemented with or without corresponding antibiotics to selectively recover the donor and recipient strains, respectively. Finally, the competitive interaction result was determined by calculating the colony count ratio of the donor strain to the recipient strain.

### 2.6. Bacterial Adhesion and Invasion Assays

The adhesion and invasion capacities were assessed using chicken embryo fibroblast DF-1 cells, as previously described [20]. DF-1 monolayers were rinsed with serum-free DMEM and challenged with bacteria at an multiplicity of infection (MOI) of 100 for 2 h at 37 °C under 5% CO_2_. Following incubation, cells were lysed with 0.5% Triton X-100, and adherent bacteria were quantified by serial dilution and plating on LB agar. For invasion assays, infected monolayers were exposed to gentamicin (100 μg/mL) in DMEM for 1 h to eliminate extracellular bacteria. After rinsing and lysis, intracellular bacteria were enumerated by plating on LB agar. Uninfected DF-1 cells served as negative controls in all experiments.

### 2.7. Determination of Bacterial Loads In Vivo

The test APEC strains were cultured to an OD_600nm_ between 0.6 and 0.8, followed by three washes with sterile PBS and final resuspension in sterile PBS. Each duckling was inoculated via the intramuscular route with a bacterial suspension containing 10^8^ colony-forming units (CFUs). At 24 h post-infection, ducklings were euthanized and dissected under aseptic conditions. Bacterial loads in the liver and spleen were quantified and expressed as CFUs per gram of tissue. Collected liver and spleen samples were homogenized and spread onto LB agar plates. Serial dilutions of the tissue homogenate were also inoculated onto LB plates to enumerate bacterial colonies. Finally, the colonization and proliferation abilities of the test APEC strains in the liver and spleen were compared.

### 2.8. Animal Infection Experiments

To investigate the impact of RelA on bacterial virulence, ducklings were divided into four groups, with 10 seven-day-old ducks in each group. Three of the groups were intramuscularly injected with 1 × 10^8^ CFUs of the bacterial strains CE08, ΔrelA, and CΔrelA, respectively. The remaining group of ducks was intramuscularly injected with an equivalent volume of PBS as negative controls. The mortality rate was monitored daily for 7 days post-infection.

### 2.9. Transcriptomic Analysis

To assess the effects of *relA* on transcriptional levels, CE08 and ΔrelA cells were collected from LB medium cultures when the OD_600nm_ reached 1.0. The cells were harvested by centrifugation at 12,000 rpm for 5 min, washed with PBS (pH 7.4), and then centrifuged again. Total RNA was isolated from pelleted bacterial cells using the Trizol RNA isolation reagent.

Ribosomal RNA was depleted from total RNA preparations using the Ribo-Zero kit to enrich for mRNA. Transcriptomic libraries derived from CE08 and the ΔrelA mutant were subsequently prepared and subjected to sequencing on the Illumina HiSeq™ 2500 or Miseq™ platform. Functional annotation of differentially expressed genes was performed through Gene Ontology (GO) enrichment and Kyoto Encyclopedia of Genes and Genomes (KEGG) pathway analysis based on the most recent database release. Read count normalization was achieved using the Trimmed Mean of M-values (TMM) algorithm, and statistical significance was evaluated under the Poisson distribution model. Genes exhibiting a fold change greater than 2 and a q-value below 0.05 were classified as differentially expressed genes (DEGs). Volcano plot analysis was employed to visualize and filter statistically significant DEGs between the wild-type CE08 and the ΔrelA strain.

### 2.10. RNA Extraction and Quantification of Gene Expression

Quantitative real-time PCR (qPCR) was performed to measure the transcriptional levels of the identified DEGs following the protocol described previously [17]. Bacterial cells grown to the logarithmic growth phase were collected for RNA extraction. Genomic DNA contamination was eliminated using the Turbo DNA-free kit (Thermo Fisher Scientific, Waltham, MA, USA), and first-strand cDNA was synthesized with the PrimeScript^®^ RT reagent kit (Takara Biomedical Technology Co., Ltd., Dalian, China) following the manufacturer’s recommended protocols. qRT-PCR was subsequently carried out to assess the transcript levels of ppGpp genes using the SYBR^®^ Premix Ex Taq™ (Takara Biomedical Technology Co., Ltd., Dalian, China). Relative gene expression levels were calculated and normalized against the housekeeping gene *dnaE* via the 2^−∆∆Ct^ method.

### 2.11. Detection of Outer Membrane Permeability and Intracellular Membrane Integrity

The outer membrane permeability of APEC was evaluated according to previously described methods [21]. Briefly, 1-N-phenylnaphthylamine (NPN) at a final concentration of 10 μM was employed as a fluorescent probe, with excitation and emission wavelengths set at 350 nm and 420 nm, respectively. Bacterial cultures were grown to OD_600nm_ of 0.6 and washed three times with HEPES buffer (pH 7.0) supplemented with 5 mM glucose. The bacterial suspension was then adjusted to an OD_600nm_ of 0.5 using the same HEPES buffer and incubated with the fluorescent dye for 1 h.

For assessment of inner membrane integrity, APEC strains were prepared following the same protocol as described for the outer membrane permeability assay. Propidium iodide (PI) was used as the fluorescent dye, with excitation and emission wavelengths of 535 nm and 615 nm, respectively [21]. Then, the bacterial solution was incubated with the fluorescent dye PI for 1 h and treated with polymyxin B as a positive control. All fluorescence measurements were performed using a microplate reader (BioTek Instruments, Inc., Winooski, VT, USA).

### 2.12. Statistical Analyses

Data entry was done using the program Microsoft Office Excel 2016. Statistical analyses were performed using GraphPad Prism version 8.0. Statistical analyses of bacterial adherence and invasion data, as well as qRT-PCR results, were performed using one-way and two-way analysis of variance (ANOVA). Comparisons between individual mutant strains and the wild-type strain were conducted via Student’s *t*-test, and differences were considered statistically significant when *p* < 0.05.

**Table 1:** Bacterial strains and plasmids used in this study.

Strains or Plasmids	Characteristics	References
**Strains**		
CE08	APEC wild-type strain	This study
ΔrelA	*relA* deletion mutant in CE08	This study
CΔrelA	ΔrelA with plasmid pSTV28-relA	This study
DH5α	Amp, with plasmid pUC19	This study
**Plasmids**		
pKD46	Amp, expresses λ red recombinase	[16]
pKD3	Cm, template plasmid	[16]
pCP20	Cm, Amp, yeast Flp recombinase gene, FLP	[16]
pSTV28-Pamp	Cm, Flag tag and His tag	[22]
pSTV28-relA	pSTV28-Pamp derivative harboring the *relA* gene	This study

**Table 2:** Primers designed and used in this study.

Primer	Primer Sequence (5′-3′)	Description
relA-mutant-F	AGTTACGATTTGCCGATTTCGGCAGGTCTGGTCCCTAAAGGAGAGGACGGTGTAGGCTGGAGCTGCTTC	Chloramphenicol resistance cassette of *relA*
relA-mutant-R	TAGATACAGTATATATCAATCTACATTGTAGATACGAGCAAGTTTCGGCCATATGAATATCCTCCTTAG	
relA-in-F	GCTGATGCCAACGTAGTCAG	Primers for identification of *relA* deletion
relA-in-R	TGCGCCAGATACTGTAGATG	
relA-out-F	AGCAGGATATACCATTGCGC	Primers for identification of *relA* deletion
relA-out-R	TATTCAGATTGAGCGCCTGC	
pSTV28-relA-F	CGCGGATCCATGGTTGCGGTAAGAAGTGCAC	Amplification of *relA* complement sequence
pSTV28-relA-R	CCCAAGCTTACTCCCGTGCAACCGACGCGCG	
clpV1-RT-F	GTGCATATCCGCACACTGGAC	Partial DNA sequence of *clpV1*
clpV1-RT-R	TGATTTCTGCACTGCATCGATG	
dotU1-RT-F	TGGAACTGGTCCGCGAACGTCTG	Partial DNA sequence of *dotU1*
dotU1-RT-R	TCGTCCATGCCACTACGGCTG	
hcp1-RT-F	CGTCAAGCTGGTCAGAGTAGC	Partial DNA sequence of *hcp1*
hcp1-RT-R	CTCGTAGCGGAACTCAATGCG	
vipA1-RT-F	CTCAAGCTTCTTGCCGTAGGC	Partial DNA sequence of *vipA1*
vipA1-RT-R	CTCAGCCATGACGCTGTTGAAG	
clpV2-RT-F	GAGACGCTCGCTACCATTATT	Partial DNA sequence of *clpV2*
clpV2-RT-R	TGATTTCGTCCGTCACTTCC	
dotU2-RT-F	ATCCTGCTCGAACGCCTGATC	Partial DNA sequence of *dotU2*
dotU2-RT-R	CATCCTGTTGCTGAATGGCAAC	
hcp2A-RT-F	CCATCAGGAGTTGACCATCACG	Partial DNA sequence of *hcp2A*
hcp2A -RT-R	GCGGTTGGTGCGGTAGAATACA	
vipA2-RT-F	ATAACACGCCGTTGGATGAGCG	Partial DNA sequence of *vipA2*
vipA2-RT-R	GTTCAGCCGGAACAACAAACTC	
CXG97_28040-RT-F	TTTCTCCTGAGCTCACTGG	Partial DNA sequence of *CXG97_28040*
CXG97_28040-RT-R	ACCACACAGAAACCAGAGAC	
CXG97_27730-RT-F	AAAGGTCCGGCATGCTATC	Partial DNA sequence of *CXG97_27730*
CXG97_27730-RT-R	ATCGTTGTCCCAGCGTTTC	
CXG97_27380-RT-F	GTGAAACGCAAGCAGTACC	Partial DNA sequence of *CXG97_27380*
CXG97_27380-RT-R	ACCGTTACCGGACATGTCC	
CXG97_27945-RT-F	TTCTTCCCGCAACTGCAATG	Partial DNA sequence of *CXG97_27945*
CXG97_27945-RT-R	GCCAGAACAACCCATTTAAC	
CXG97_27750-RT-F	AACAACTGTGGCCGAAAGAC	Partial DNA sequence of *CXG97_27750*
CXG97_27750-RT-R	GCCATTTAATGCTGGAAGTC	
CXG97_27660-RT-F	ACAATGAATGCCACCTCTGG	Partial DNA sequence of *CXG97_27660*
CXG97_27660-RT-R	CCACTCTGAAGGGATAAAAC	
CXG97_27515-RT-F	ATTGCGTCCATGCGTATCC	Partial DNA sequence of *CXG97_27515*
CXG97_27515-RT-R	ACCAGTAAATACAGCCGTTC	
CXG97_27605-RT-F	TTGCTCCTCTTCTCCATCC	Partial DNA sequence of *CXG97_27605*
CXG97_27605-RT-R	ACAGAGACGACCTTTGTCTC	
CXG97_27950-RT-F	CTGCCACTGGATGAACTG	Partial DNA sequence of *CXG97_27950*
CXG97_27950-RT-R	TACCAGTAAATCAGGTCACG	
CXG97_27955-RT-F	GCCCGTTTAAGTACCAGTC	Partial DNA sequence of *CXG97_27955*
CXG97_27955-RT-R	TCTGTGACACGGCAAAGGG	
CXG97_27610-RT-F	ATAGAAGGCAAGGCGTTCG	Partial DNA sequence of *CXG97_27610*
CXG97_27610-RT-R	TTCAATGCACGGGTCTGATC	
CXG97_27370-RT-F	GAACACGGTGCCCGTTTAAG	Partial DNA sequence of *CXG97_27370*
CXG97_27370-RT-R	GTCGGCACTGTTCTGTGAC	

## 3. Results

### 3.1. Effect of RelA Inactivation on APEC Growth and Motility

**Figure 1:**
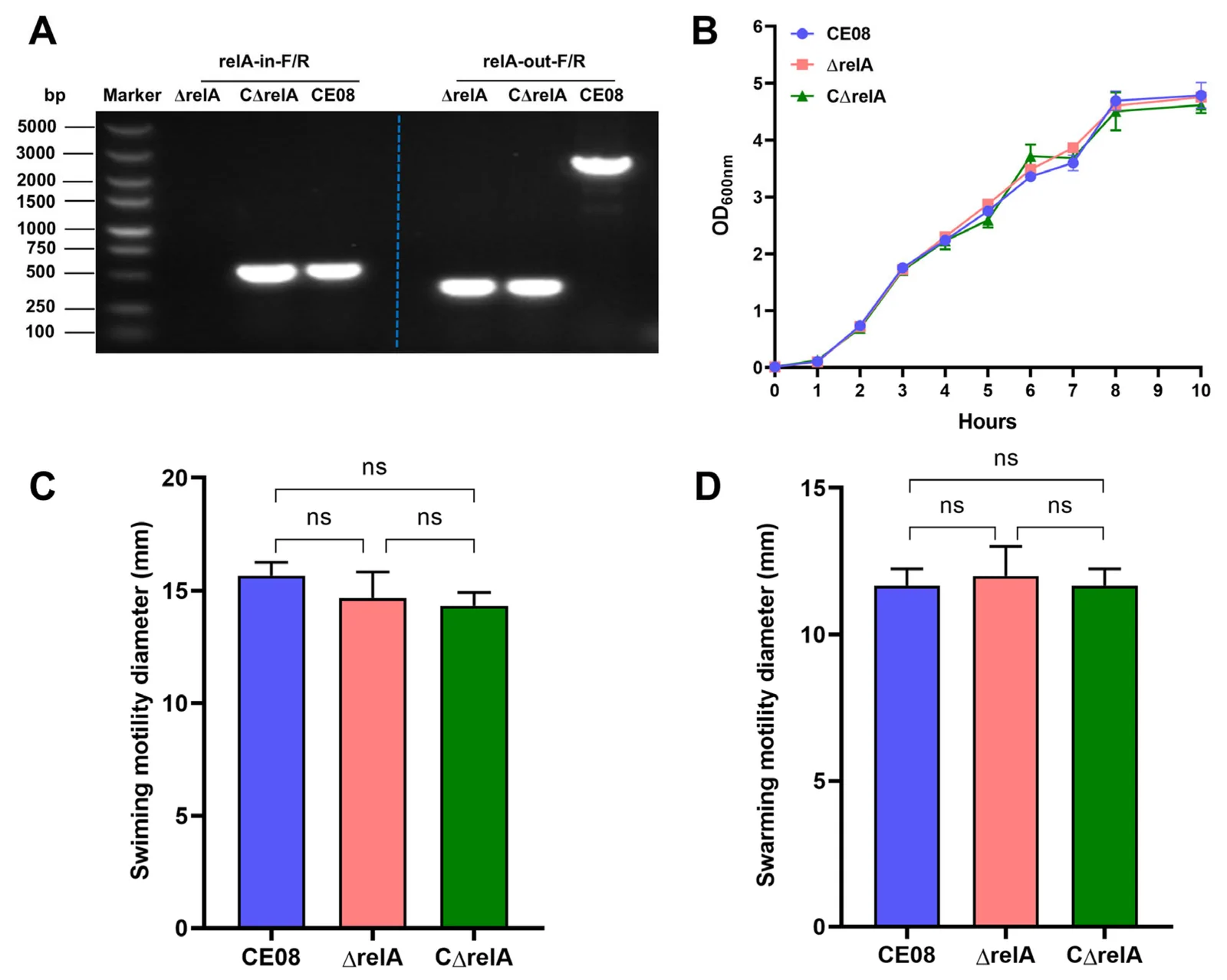
Biological characterization of the test APEC strains. (**A**) PCR identification results of the ΔrelA mutant and CΔrelA complemented strain. (**B**) Bacterial growth curves. All strains were cultured in LB medium at 37 °C, and optical density at 600 nm was measured hourly. (**C**) Bacterial swimming motility. The bacterial motility haloes in LB swimming (0.3% agar) assays were measured after overnight incubation at 37 °C. (**D**) Bacterial swarming motility. The bacterial motility haloes in LB swarming (0.5% agar) assays were measured after overnight incubation at 37 °C. Significant differences were analyzed using one-way ANOVA (ns, *p* > 0.05).

In this study, we constructed and genetically characterized the *relA* gene mutant and complementary strains of APEC. Both the ΔrelA mutant and CΔrelA complemented strain were successfully verified via PCR (Figure 1A). No PCR product was amplified from the ΔrelA mutant with the relA-in-F/relA-in-R primer pair, whereas a specific product was detected in CE08 and CΔrelA. With the relA-out-F/relA-out-R primer pair, a PCR product of expected size was amplified in CE08, and a smaller fragment was obtained for ΔrelA and CΔrelA. As seen in Figure 1B, the growth curve showed that the mutant strain ΔrelA grew similarly to the wild-type CE08 and complementation strain CΔrelA during 10 h. These results showed that the deletion of the *relA* gene had no evident effect on the growth of APEC under normal conditions. The examination of bacterial migration showed that there was no significant difference (*p* > 0.05) in halo diameter on swimming (Figure 1C) and swarming (Figure 1D) agar plates among the APEC strains. These results show that deleting *relA* does not significantly affect swimming or swarming in CE08 under normal conditions.

### 3.2. Inactivation of *RelA* Effects on Antibiotic Resistance of APEC

The MIC of various aminoglycosides for the CE08, ΔrelA, and CΔrelA strains were determined (Table 3). The MIC of streptomycin was 128 μg/mL against CE08, which decreased to 64 μg/mL for both ΔrelA and CΔrelA strains. For kanamycin, MIC values were 16, 4, and 8 μg/mL for CE08, ΔrelA, and CΔrelA strains, respectively. The MIC of gentamicin against CE08 was 2 μg/mL, and the MIC for ΔrelA and CΔrelA was 1 μg/mL. The MIC of spectinomycin was 128 μg/mL for CE08 and CΔrelA, and 64 μg/mL for ΔrelA. These results indicate that *relA* deletion significantly increases susceptibility to several aminoglycosides.

### 3.3. The *RelA* Effects on Interbacterial Competition of APEC

**Figure 2:**
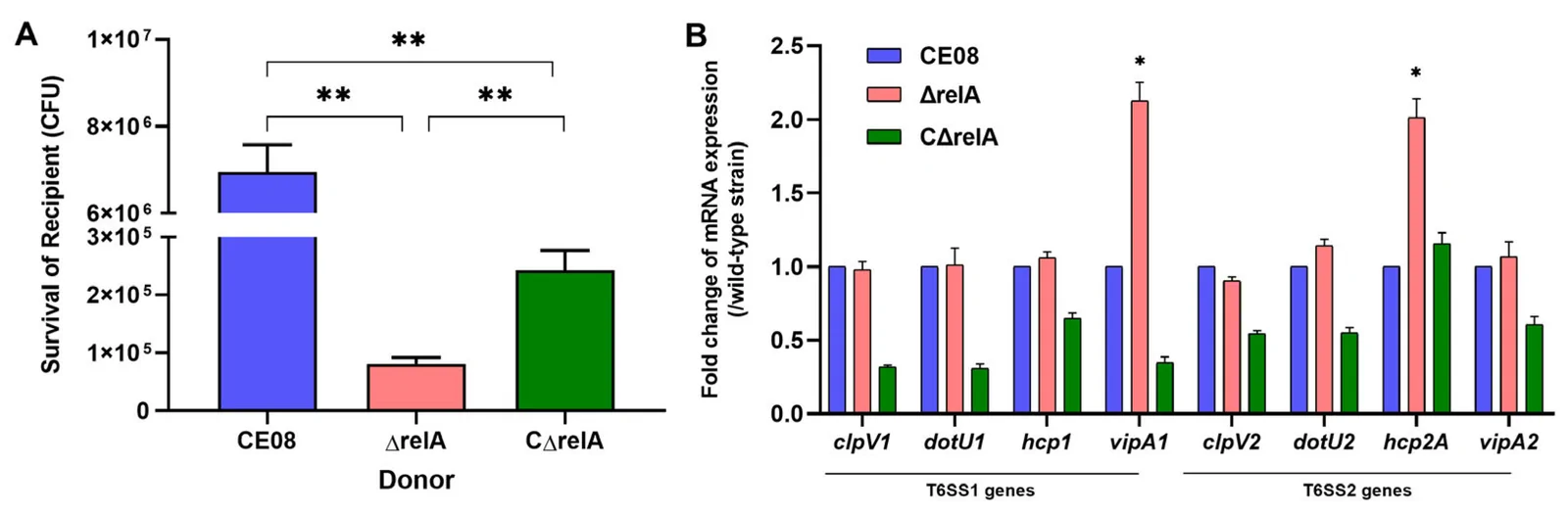
RelA modulates interbacterial competition and T6SS gene expression in APEC. (**A**) Competitive fitness of APEC strains against *E. coli* DH5α. Freshly cultured APEC donors were co-incubated with DH5α recipients at a 5:1 ratio for 6 h at 30 °C. The ΔrelA mutant exhibited significantly enhanced competitive ability relative to both the wild-type and complemented strains. (**B**) Transcriptional profiling of T6SS-associated genes. qRT-PCR analysis of *vipA1* and *hcp2A* transcript levels in wild-type, ΔrelA, and CΔrelA strains, normalized to the housekeeping gene *dnaE*. Data are expressed as fold changes relative to the wild-type. Statistical analyses were performed using one-way ANOVA or two-way ANOVA, as appropriate (*, *p* < 0.05; **, *p* < 0.01).

To further elucidate the role of RelA in interbacterial competition, we conducted co-culture assays using freshly grown APEC strains as donors and *E. coli* DH5α as the recipient, then enumerated the survivors. When ΔrelA served as the donor, DH5α viability was markedly reduced (*p* < 0.01, Figure 2A). This indicates that the ΔrelA mutant possesses stronger antimicrobial competition than both the wild-type and complemented strains. To explore whether this enhanced activity is linked to alterations in the type VI secretion system (T6SS), we performed qPCR to analyze the expression of T6SS core genes in the tested APEC strains. Our results demonstrated that deletion of the *relA* gene significantly upregulated the transcription of two core T6SS genes, *vipA1* and *hcp2A* (*p* < 0.05; Figure 2B).

### 3.4. *RelA* Contributes to Bacterial Adhesion and Invasion to DF-1 Cells

**Figure 3:**
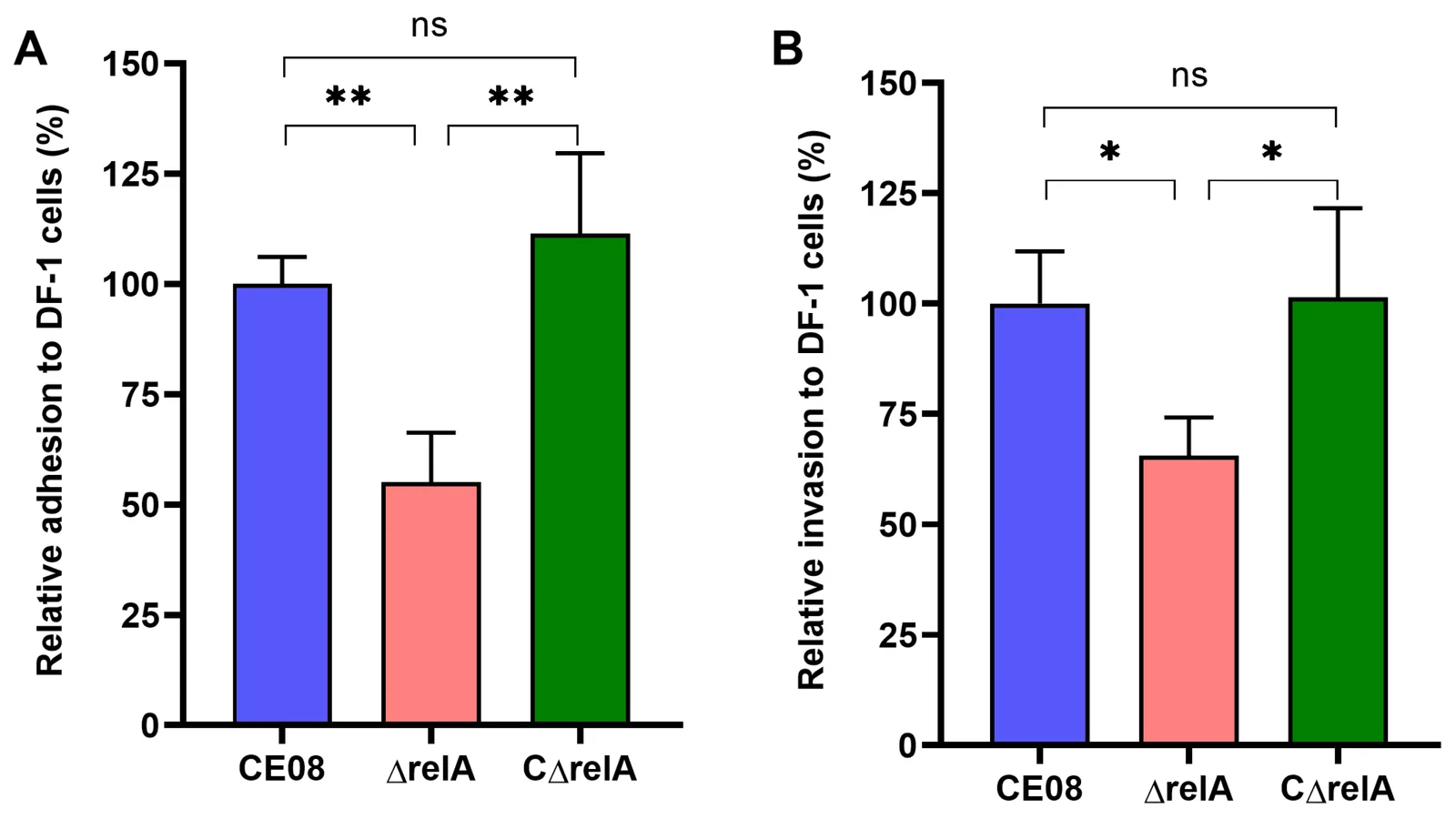
Adhesion and invasion assays of APEC strains. (**A**) Assessment of the adhesive capacity of APEC to DF-1 cells. Compared with the wild-type and complemented strains, the ΔrelA mutant showed significantly lower adhesion ability to DF-1 cells. (**B**) Quantification of the invasive capacity of APEC into DF-1 cells. The ΔrelA mutant exhibited a marked reduction in invasive capacity relative to the wild-type and complemented strains. All statistical significance analyses were conducted using one-way ANOVA (ns, *p* > 0.05; *, *p* < 0.05; **, *p* < 0.01).

We analyzed differences in adhesion and invasion capacity among wild-type, *relA* mutant, and complemented APEC strains. Our results demonstrated that, compared with the wild-type strain, the mutant exhibited a significant reduction in adhesion and invasion ability toward DF-1 cells (Figure 3A,B). The complemented strain restored the adhesion and invasion capacities. These results showed that the *relA* gene promotes bacterial adhesion and invasion.

### 3.5. *RelA* Plays a Critical Role in Bacterial Survival and Virulence In Vivo

**Figure 4:**
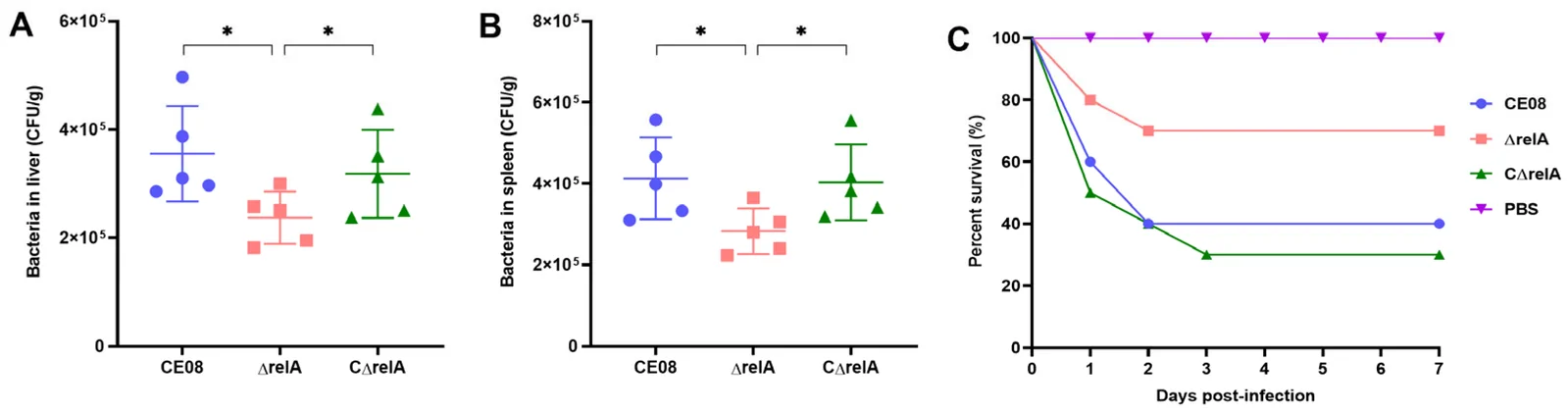
RelA contributes to the colonization and virulence of APEC. (**A**) Bacterial loads in liver. Ducks were challenged with different APEC strains and were euthanized at 24 h post-infection to isolate and quantify bacteria from liver tissues. (**B**) Bacterial loads in spleen. Bacteria were isolated and quantified from spleen tissues collected from euthanized infected ducks at 24 h post-infection. (**C**) Survival curve analysis. After challenge with the indicated APEC strains, duck mortality was recorded daily for 7 days post-infection; ducks injected with PBS were included as negative controls. Statistical significance was analyzed using the non-parametric Mann–Whitney U test (*, *p* < 0.05).

To explore the functional role of RelA in APEC pathogenicity, we assessed the virulence of tested APEC strains using a duck infection model. To further examine whether RelA influences APEC colonization during systemic infection, we quantified bacterial loads in the liver and spleen at 24 h post-infection. The ΔrelA mutant exhibited significantly reduced colonization in both the liver and spleen compared to wild-type CE08 (*p* < 0.05) (Figure 4A,B). The complemented strain showed a significant difference from the mutant and restored the same level as the wild-type strain. These results showed that RelA is required for effective APEC colonization and survival during systemic infection.

Survival curve analysis revealed that the wild-type strain CE08 resulted in higher duck mortality than the ΔrelA mutant strain (Figure 4C). Specifically, the mortality of ducklings infected with CE08, ΔrelA, and the complemented strain CΔrelA was 60% (6/10), 30% (3/10), and 70% (7/10), respectively. Collectively, these results confirm that deletion of the *relA* gene attenuates APEC virulence in ducks, and the attenuated virulence can be fully restored in the complementation strain.

### 3.6. Analysis of Screening Differential Transcriptional Genes Regulated by relA Based on RNA-Seq Technology

**Figure 5:**
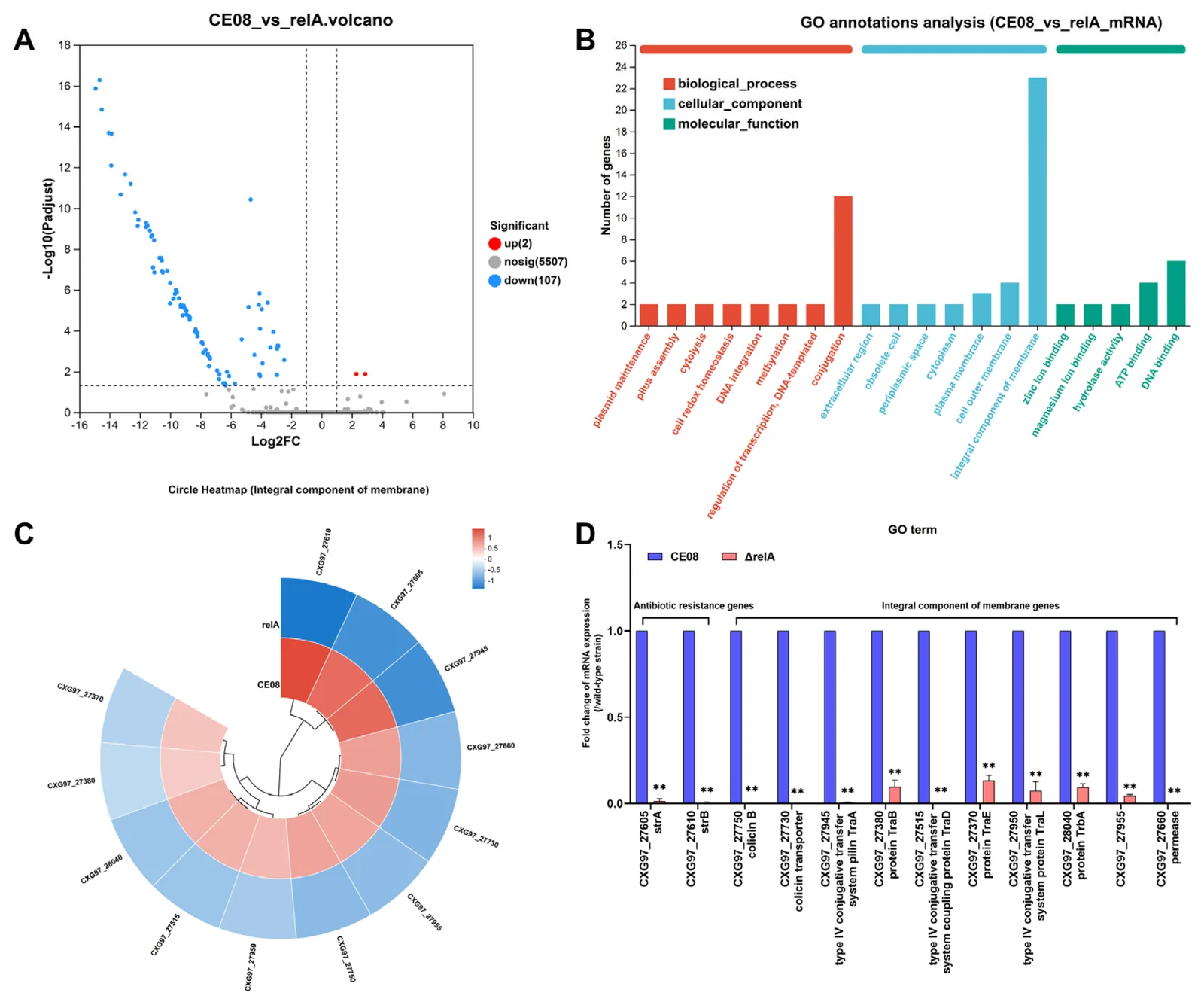
Transcriptomic profiling of the ΔrelA mutant strain. (**A**) Volcano plot of differentially expressed genes (DEGs) between the wild-type strain CE08 and mutant ΔrelA. (**B**) GO annotation analysis of DEGs. (**C**) Circular heatmap of DEG expression levels. (**D**) Experimental validation of selected DEGs by RT-qPCR. Statistical significance analysis was performed by one-way ANOVA or two-way ANOVA, respectively (**, *p* < 0.01).

Differential gene expression between the wild-type strain CE08 and the mutant ΔrelA was analyzed using strand-specific Illumina RNA-Seq. Transcriptomic analysis identified 107 downregulated and two upregulated genes in the ΔrelA mutant relative to the wild-type strain (Figure 5A). The downregulated genes included those involved in antibiotic resistance and membrane composition. Gene Ontology (GO) enrichment analysis showed that these downregulated genes were significantly associated with the term integral component of the membrane (Figure 5B). Some genes within this group showed significant downregulation, as shown in the heatmap (Figure 5C). Furthermore, the downregulated expression of these 12 genes was validated by real-time quantitative PCR (Figure 5D).

### 3.7. *RelA* Affects Outer Membrane Permeability and Inner Membrane Integrity of APEC

**Figure 6:**
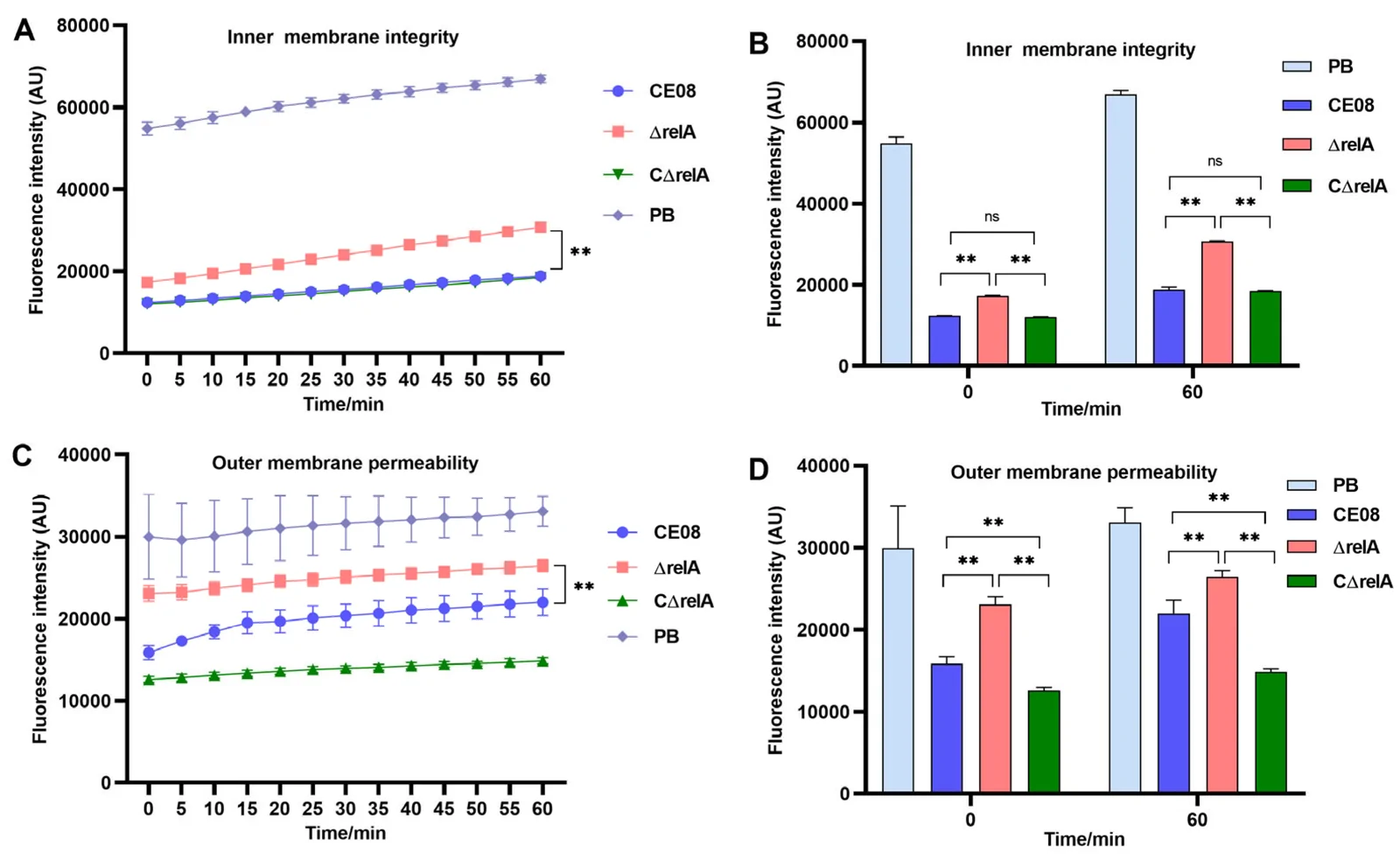
Determination of outer membrane permeability and inner membrane integrity. (**A**) Continuous monitoring of inner membrane integrity (0–60 min). (**B**) Changes in inner membrane integrity at 60 min. The integrity of the inner membrane of APEC was detected by the PI dye. (**C**) Continuous monitoring of outer membrane permeability (0–60 min). (**D**) Changes in outer membrane permeability at 60 min. The NPN probe was used to detect the outer membrane permeability of APEC. Significant differences were determined by one-way ANOVA (ns, *p* > 0.05; **, *p* < 0.01).

Since deletion of *relA* altered the transcription of membrane component genes in APEC, we further investigated its impact on inner and outer membrane integrity. NPN and PI were employed to detect alterations in the outer membrane and inner membrane, respectively. Polymyxin treatment, which served as the positive control, disrupted the integrity of both outer and inner membranes of strain CE08. The results demonstrated that the deletion of the *relA* gene significantly disrupted inner membrane integrity and increased outer membrane permeability in APEC (Figure 6A,B for inner membrane; Figure 6C,D for outer membrane). In contrast, these membrane defects were restored in the complemented strains.

## 4. Discussion

APEC is a significant pathogen in the global poultry industry, capable of causing respiratory tract lesions, septicemia, and other clinical conditions that lead to high mortality rates in birds and substantial economic losses. APEC exhibits remarkable environmental adaptability, employing mechanisms such as the stringent response to cope with stresses such as nutrient deprivation and osmotic pressure changes. Within the host, APEC utilizes adhesion and invasion factors to colonize tissues such as the liver, evade host immune clearance, and poses a serious threat to animal health and food safety. In *Escherichia coli*, the enzyme RelA is activated during amino acid starvation and synthesizes (p)ppGpp. This alarmone mediates the stringent response, a process that allows the bacterium to adapt to environmental stresses including nutrient limitation, antibiotic exposure, and host conditions [9]. While the role of (p)ppGpp in virulence and antibiotic resistance has been studied in various pathogens, its function in APEC remains poorly understood. Our study demonstrates that RelA, a key (p)ppGpp synthetase, is not essential for in vitro growth or motility but significantly contributes to APEC virulence, antibiotic resistance, interbacterial competition, and membrane integrity.

The absence of RelA did not affect APEC growth or motility under standard laboratory conditions. However, RelA deletion markedly increased APEC susceptibility to aminoglycosides such as streptomycin, kanamycin, gentamicin, and spectinomycin. This aligns with previous reports that (p)ppGpp can modulate antibiotic resistance through multiple mechanisms, including efflux pump expression, membrane permeability, and metabolic reprogramming [23,24,25]. For some aminoglycosides (e.g., streptomycin, gentamicin), the complemented strain did not fully restore the wild-type MIC. This incomplete restoration may be attributed to polar effects or plasmid-based expression limitations. In *Salmonella*, (p)ppGpp upregulates aminoglycoside adenyl transferase expression, conferring resistance to streptomycin and spectinomycin [26]. Our findings suggest that RelA-mediated (p)ppGpp synthesis may similarly regulate resistance determinants in APEC, though the exact targets require further investigation. Transcriptomic analysis revealed that RelA deletion downregulated numerous genes involved in membrane integrity, including several encoding integral membrane proteins. This was corroborated by increased outer membrane permeability and inner membrane disruption in the ΔrelA mutant, as assessed by NPN and PI uptake assays. Membrane integrity is crucial for antibiotic resistance, stress survival, and virulence [27,28]. In *E. coli*, (p)ppGpp functions as a critical coordinator specifically induced by disruptions in outer membrane biogenesis and ADP synthesis. It orchestrates a growth-arresting stress response to promote survival, while (p)ppGpp-deficient cells lyse from uncontrolled proliferation. These findings underscore a novel, essential role for (p)ppGpp in maintaining membrane integrity and metabolic homeostasis [29]. Our findings suggest that RelA is involved in maintaining membrane stability in APEC, which may represent the primary mechanism through which RelA influences antibiotic sensitivity and virulence. RelA maintains membrane integrity, which directly affects antibiotic influx. In APEC, RelA-mediated (p)ppGpp likely synergizes the classic persister pathway (metabolic reprogramming) and membrane homeostasis to achieve antimicrobial resistance.

A key virulence phenotype affected by RelA deletion was the reduced adhesion to and invasion of DF-1 cells. This is consistent with studies of enteropathogenic *E. coli*, where (p)ppGpp activates the LEE pathogenicity island via Ler and Pch to enhance host cell adhesion and invasion [10]. Similarly, in uropathogenic *E. coli* (UPEC), it promotes adhesion by inducing type 1 fimbriae expression via FimB [12]. In *Salmonella*, (p)ppGpp is required for invasion gene expression and systemic infection [14]. Our in vivo duck infection model confirmed that RelA is essential for APEC virulence, as the ΔrelA mutant showed attenuated colonization in the liver and spleen and reduced mortality. These findings demonstrate the important role of (p)ppGpp in facilitating APEC adaptation and survival within the host.

T6SS1 and T6SS2 are critical for APEC pathogenicity and interbacterial competition [30]. This study found that the deletion of *relA* enhanced the interbacterial competition of APEC. However, the upregulated transcriptional levels of the core component genes of T6SS1 and T6SS2 may account for the increased competitive capacity of the ΔrelA mutant. The *vipA1* and *hcp2A* genes play distinct yet critical roles in mediating T6SS structural assembly and effector delivery. As a key structural subunit of the T6SS1 contractile sheath, VipA1 polymerizes with VipB1 to form a dynamic, cogwheel-like sheath complex [31]. Upon activation, the contraction of this sheath propels the Hcp1-based inner tube and VgrG spike into adjacent bacterial or eukaryotic cells. This process enables the injection of virulence effectors that are essential for bacterial competition and host cell invasion. The Hcp2A is a major structural component of the T6SS2 inner tube, which self-assembles into a tubular structure to transport antibacterial and host-targeting effectors [32]. In APEC, Hcp2A directly interacts with the host ribosomal protein RPL23, a mechanism that enhances host cell invasion and promotes systemic virulence, thereby contributing to bacterial virulence [33]. Collectively, *vipA1* underpins the functional integrity of T6SS-1 via sheath formation, while *hcp2A* not only forms the T6SS-2 secretion conduit but also mediates direct host cell interactions, making both genes pivotal for the pathogenicity of *E. coli* in animal and human hosts. The upregulation of T6SS in ΔrelA may represent a compensatory stress response to membrane defects and (p)ppGpp depletion. While this enhances antibacterial competition in vitro, it cannot compensate for the loss of adhesion factors and membrane integrity required for host colonization, resulting in net attenuation of virulence.

In conclusion, RelA plays a multifaceted role in APEC pathogenesis, influencing antibiotic resistance, bacterial competition, host cell adhesion, systemic colonization, and membrane integrity. These findings provide a critical foundation for comprehensively understanding the pathogenic mechanisms of APEC.

## 5. Conclusions

In summary, this study systematically characterized the biological functions of the (p)ppGpp synthetase RelA in avian pathogenic *E. coli* (APEC). Loss of *relA* had no obvious influence on APEC growth and motility under standard culture conditions, but disrupted the inner and outer bacterial membrane integrity, which increased susceptibility to multiple aminoglycoside antibiotics. Notably, complementation restored resistance only partially for some antibiotics. Deletion of *relA* also upregulated core T6SS genes, enhancing the interbacterial competitive capacity of the mutant. Moreover, RelA was required for efficient adhesion and invasion of chicken DF-1 cells, as well as tissue colonization and full virulence in duckling infection models. Transcriptomic profiling further confirmed that RelA globally modulates the expression of membrane component genes, which acts as the core mechanism linking the stringent response to APEC antibiotic resistance and pathogenicity. Collectively, our findings demonstrate that the RelA-mediated stringent response maintains membrane homeostasis to coordinate APEC stress adaptation and virulence. This work expands the understanding of (p)ppGpp signaling in avian pathogenic bacteria and provides a potential target for developing novel strategies to prevent and treat avian colibacillosis.

## Figures and Tables

**Table 3:** The results of the minimum inhibitory concentration.

Antibiotic	MIC (μg/mL)		
	CE08	ΔrelA	CΔrelA
Streptomycin	128	64	64
Kanamycin	16	4	8
Gentamicin	2	1	1
Spectinomycin	128	64	128

## Data Availability

The original contributions presented in this study are included in the article. Further inquiries can be directed to the corresponding author.
